# Unveiling cancer crosstalk: Mapping complexity across time and space

**DOI:** 10.1371/journal.pbio.3003326

**Published:** 2025-07-29

**Authors:** Yibin Kang

**Affiliations:** 1 Department of Molecular Biology, Princeton University, Princeton, New Jersey, United States of America; 2 Ludwig Institute for Cancer Research Princeton Branch, Princeton, New Jersey, United States of America

## Abstract

Cancer evolves through dynamic exchanges with its environment, harnessing these interactions to grow, adapt and transcend the constraints that would otherwise limit its progress. This Editorial introduces a new collection of articles that explore this tumor–environment crosstalk across temporal and spatial scales.

## Teaser

Cancer evolves through dynamic exchanges with its environment, harnessing these interactions to grow, adapt, and transcend the constraints that would otherwise limit its progress. A new collection of articles explores this tumor–environment crosstalk across temporal and spatial scales.

Cancer is not a singular disease but a complex and evolving system. As tumors grow and spread, they engage in continuous and reciprocal communication with surrounding tissues, immune cells, stromal components, and even distant organs [1,2]. These interactions are dynamic, occurring across both spatial landscapes (within and between tissues) and temporal scales (from tumor initiation to metastatic colonization). With the advent of spatially resolved and temporally sensitive technologies, researchers are now able to map this crosstalk in unprecedented detail.

This issue of *PLOS Biology* features a collection of forward-looking Essays and Perspectives that explore the intricate choreography between cancer cells and their microenvironments. The articles provide insights into how emerging tools in single-cell analysis, spatial omics, computational modeling, and metabolic profiling are reshaping our understanding of tumor biology. From evolutionary theory to clinical translation, each contribution offers a distinctive angle on the multifaceted dialogue that underpins cancer progression.

In their Essay on multilevel selection, Laplane and colleagues argue that cancer cannot be fully understood without considering evolutionary pressures operating at multiple biological scales—from genes and organelles to cells and organisms [3]. By reframing cancer as a phenomenon shaped by selection across levels, the authors provide a conceptual foundation for examining how competing units of selection influence tumor development and suppression.

Munn and Jain turn to computational oncology, illustrating how mechanistic and artificial intelligence (AI)-driven tumor models are becoming indispensable tools for simulating complex biological systems [4]. These models integrate molecular and physiological data to capture the emergent behavior of tumors within their microenvironments, offering a platform for hypothesis testing and therapeutic prediction in silico.

The power of spatial and single-cell omics is fully demonstrated by Liu and Zhang, who examine how metastatic niches are formed and maintained [5]. Their Essay reveals how disseminated tumor cells interact with immune and stromal cells at distant sites, undergo metabolic reprogramming and evade immune surveillance; all processes made visible through high-resolution, multimodal profiling.

In a complementary Perspective, Huang and colleagues discuss how large-scale omics datasets are being translated into clinically actionable tools [6]. From target discovery to the refinement of molecular subtypes and predictive modeling, they show how integrated omics is bridging the longstanding gap between tumor biology and patient care.

Expanding the spatial theme further, Sloan and Lee spotlight the tumor neural niche, a relatively understudied component of the tumor microenvironment [7]. They detail how autonomic and sensory nerves interact with cancer and immune cells, modulating invasion, immune evasion and treatment response. Their Perspective highlights innervation as a novel, targetable axis in cancer progression.

Mo and colleagues issue a call to reimagine the tumor not as a mass of transformed cells, but as a diseased tissue shaped by complex multicellular ecosystems [8]. Their Perspective emphasizes the critical roles of cancer-associated fibroblasts and extracellular matrix remodeling in therapy resistance, immune exclusion and tumor heterogeneity. By centering the tumor microenvironment in both research and drug development, they argue for a necessary shift in therapeutic strategy.

On the metabolic front, Li and colleagues delve into the bi-directional reprogramming that occurs between cancer cells and the immune system [9]. They explain how tumors outcompete T cells for key nutrients, secrete immunosuppressive metabolites and adapt to hypoxic and acidic conditions, while T cells, in turn, attempt to rewire their own metabolism to maintain effector functions. These interactions are framed as central to both tumor survival and therapeutic resistance.

Finally, Rosenbaum, Fields and Ford examine the theme of mutual plasticity, in which both cancer and immune cells adapt in response to one another [10]. The authors explore how epithelial-to-mesenchymal transition, immune editing and dynamic immune suppression contribute to tumor evolution and heterogeneity. They also suggest that such plasticity may be exploited for therapeutic gain, particularly in designing treatments that account for cellular states and their transitions.

Together, these articles reflect a growing consensus that understanding cancer requires an integrated view, one that goes beyond static snapshots and reductionist models. The tools highlighted in this collection, including spatial transcriptomics, lineage tracing, AI-enabled simulations and metabolic flux analysis, are enabling this systems-level approach. Importantly, many of these technologies are not only descriptive but predictive, holding the potential to guide new strategies in diagnostics, prognostics and personalized therapy.

The articles in this collection also reveal convergent themes across diverse domains: the importance of cellular context; the impact of spatial organization and temporal dynamics; and the value of studying cancer as a living, adapting system. These insights are not only conceptually unifying but also practically transformative, offering new avenues for research and intervention.

As the field moves forward, we face key questions. How can we integrate these rich datasets into coherent models of disease? Can we capture and target the temporal dynamics of cancer plasticity? How might therapies be adapted to account for spatial heterogeneity or metabolic crosstalk? Answering these questions will require continued interdisciplinary collaboration across genomics, computation, evolutionary biology and clinical oncology.

This collection serves not only as a snapshot of where the field stands but also as a springboard for future inquiry. By unveiling the crosstalk that drives cancer across space and time, we edge closer to understanding—and ultimately outmaneuvering—one of biology’s most complex adversaries.
